# A Retrospective Study of Lenvatinib Monotherapy or Combined With Programmed Cell Death Protein 1 Antibody in the Treatment of Patients With Hepatocellular Carcinoma or Intrahepatic Cholangiocarcinoma in China

**DOI:** 10.3389/fonc.2021.788635

**Published:** 2021-12-17

**Authors:** Sihui Zhu, Chenxi Liu, Yanbing Dong, Jie Shao, Baorui Liu, Jie Shen

**Affiliations:** ^1^ Comprehensive Cancer Centre of Drum Tower Hospital, Medical School of Nanjing University, Clinical Cancer Institute of Nanjing University, Nanjing, China; ^2^ Comprehensive Cancer Centre of Nanjing Drum Tower Hospital, Clinical College of Nanjing Medical University, Nanjing, China

**Keywords:** lenvatinib, hepatocellular carcinoma, intrahepatic cholangiocarcinoma, anti-PD-1, progression-free survival, overall survival

## Abstract

Lenvatinib has been ratified as a first-line medication for advanced liver tumors by the American Food and Drug Administration. To assess the effectiveness and security of Lenvatinib in the Chinese population in a real-world setting, we enrolled 48 patients with unresectable liver cancer, managed from December 2018 to March 2021. Among them, 9 and 39 (83.30% men) patients had intrahepatic cholangiocarcinoma (ICC) and hepatocellular carcinoma (HCC), respectively. Twenty-one (43.75%) patients had progressive disease after first-line treatment, and others (56.25%) had not receiving systemic treatment. Lenvatinib was administered alone or in combination with a programmed cell death protein 1 antibody (anti-PD-1). Treatment duration, median progression-free survival (mPFS), and median overall survival (mOS) were examined. The mOS and mPFS were 22.43 and 8.93 months, respectively. Of HCC patients treated with Lenvatinib only, the mOS and mPFS were 22.43 and 11.60 months, respectively. The corresponding values for HCC cases managed with anti-PD-1 combined with Lenvatinib were 21.77 and 7.10 months, respectively. ICC patients did not reach the mOS and their mPFS was 8.63 months. The present findings support the efficacy and security of Lenvatinib in the real-world therapy of Chinese patients with unresectable liver cancer.

## Introduction

There were an estimated 905,700 new cases of liver cancer, and approximately 830,200 associated deaths in 2020 worldwide (1). Globally, liver cancer has being the second most common causation of cancer-related deaths (1).

Sorafenib, a multikinase inhibitor that targets raf, platelet-derived growth factor, vascular endothelial growth factor, and tyrosine kinases (2, 3) was made offical by the Food and Drug Administration in November 2007 for the treatment of advanced liver cancer and it remained the standard of care for over a decade, prolonging the median survival of patients in clinical trials (4). In August 2018, data from the Phase III REFLECT trial (NCT01761266) have enabled the same agency to approve another small-molecule tyrosine kinase inhibitor as first-line medication of advanced liver cancer, Lenvatinib, which inhibits the activity of the vascular endothelial growth factor receptor, platelet-derived growth factor receptor α, stem cell factor receptor, fibroblast growth factor receptor, and rearrangement during transfection (5, 6). However, the median survival time of patients receiving Lenvatinib monotherapy was 13.6 months in a clinical trial (7).

ICIs are monoclonal antibodies that can block the interaction between immune checkpoint proteins and their ligands, thereby enhancing the anti-tumor immune response by preventing T cell inactivation and restoring immune recognition and immune attack. At present, it mainly includes anti-PD-1, anti-PD-L1 and anti-CTLA-4 (8). Anti-PD-1 can bind to its ligands PD-L1 or PD-L2 which expressed in various tumors, including HCC (9). It was found that pembrolizumab treatment shown promising clinical effects in patients with advanced hepatocellular carcinoma after sorafenib treatment fails in KEYNOTE-224 clinical trial. The mPFS is 4.9 months and the median overall survival is 12.9 months in this trial (10). In 2017, the PD-1 inhibitor nivolumab received accelerated approval in the United States for the second-line treatment of patients with advanced HCC after sorafenib treatment (11).

Combination of immunotherapy strategies are being developed to enhance liver tumor response to immune checkpoint inhibitors (12). The combination of tyrosine kinase inhibitors or vascular endothelial growth factor inhibitors and immune checkpoint inhibitors may enhance dendritic cell and cytotoxic T lymphocyte activity and inhibit tumor-associated macrophage, regulatory T-cell, and myeloid-derived suppressor cell regulation of the immune microenvironment, thereby creating an inflammatory microenvironment associated with relatively effective and long-lasting responses to checkpoint inhibitors (13). In a phase Ib trial (KEYNOTE-524), the mPFS and mOS of HCC patients treated with Lenvatinib and anti-PD-1 were 8.6 and 22 months, respectively (14).

However, clinical trials may not fully reflect real-world treatment efficacy, while risk factors for liver cancer may differ among populations (15). In China, the dominating causation of HCC is chronic hepatitis B virus infection. While the major risk factor for HCC in developed countries is nonalcoholic fatty liver disease (16). Furthermore, clinical trials tend to exclude patients with clear invasion into the bile duct or main portal vein. Patients who had received systemic treatment such as chemotherapy or sorafenib tend to be also excluded (7). Therefore, this retrospective study comprised 48 patients with HCC or ICC treated with Lenvatinib alone or combined with anti-PD-1, aiming to appraise the efficacy and safety of these treatments in clinical practice in China.

## Materials and Methods

We included 56 cases with unresectable liver cancer treated with Lenvatinib from December 2018 to March 2021 at the Comprehensive Cancer Center of Drum Tower Hospital of Nanjing University. There were 48 patients analyzable and well-documented. A total of 21 HCC patients were managed with Lenvatinib only, while a total of 18 and 9 patients with HCC and ICC, respectively, were treated with combination therapy. The patients’ average age was 59.7 years. Eight patients were female; 6 cases of them were HCC and other 2 cases were ICC. Twenty-one (43.75%) patients had progressive disease after receiving first-line treatment; meanwhile, 27 (56.25%) patients received no systemic treatment. According to the Child-Pugh score, 4 patients had grade B liver function, of which 3 were HCC; the remaining patients had grade A liver function. Forty patients had cirrhosis at baseline, including all ICC cases and 31 HCC cases. There were 4 ICC patients and 35 HCC patients had hepatitis B virus infection, respectively. Most patients had tumor node metastasis (TNM, American Joint Commission on Cancer 8th edition) stage IV; only 11 patients had stage III disease, and all of them were HCC cases. In addition, in HCC patients, 39 cases all presented with microvascular invasion; 33 cases had tumor invasion into the macroscopic portal vein or extrahepatic spread, or both; 12 cases had lung metastases. Only one HCC patient had no liver lesions. Six HCC patients were classified as Barcelona Clinic Liver Cancer stage B; the remaining patients were classified as stage C. The specific characteristics of HCC patients are presented in **Supplementary Table 1**.

### Treatment Measures

A total of 21 patients with HCC were managed with Lenvatinib only (8 mg daily), as they were in good general condition. Twenty-seven patients received combined treatment, including 18 cases with HCC and other 9 cases with ICC (Lenvatinib plus anti-PD-1 treatment at a dose of 8 mg daily and 200 mg every 3 weeks, respectively). These patients tended to be in later stage of disease than their monotherapy counterparts, including larger tumor burden, more advanced disease stage, and pathology findings indicative of poorer prognosis.

### Study Design

This retrospective cohort study was founded on medical records of patients with unresectable liver cancer, undergoing the treatment of interest. Disease progression and mortality were examined; the primary outcome was PFS, defined as the time from medication inaugural to tumor progression or death (clinical response was evaluated with the Response Evaluation Criteria in Solid Tumors). The minor endpoints involved OS, defined as the time from medication inaugural to death, objective remission rate (ORR), and performance status scores. Safety assessment evaluation included vital signs, and blood and biochemical examination findings, among others. Adverse events were recorded based on the National Cancer Institute Common Terminology Criteria for Adverse Events.

### Data Analysis

The Kaplan-Meier method was used to estimate time-to-event outcomes. All statistical analyses were performed by Graphpad 7.0. P-values of < 0.05 were considered statistically significant.

## Results

**Figure 1:**
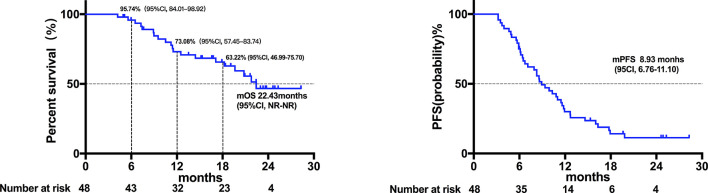
Kaplan-Meier estimates of overall and progression-free survival in all patients. The mOS of all patients was 22.43 months (95% confidence interval was not reached; range: 4.20-28.30 months), and the mPFS of all patients was 8.93 months (95% CI, 6.76-11.10; range, 3.20-28.30 months).

**Figure 2:**
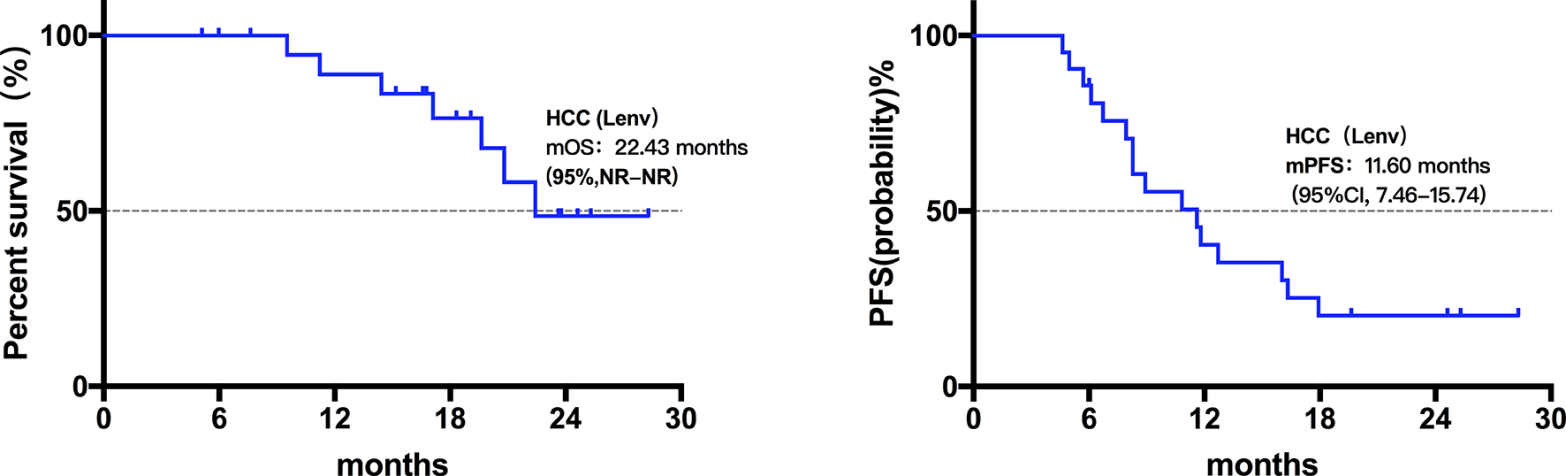
Kaplan-Meier estimates of overall and progression-free survival in HCC patients treated with Lenvatinib monotherapy. The mOS of HCC patients treated with Lenvatinib only was 22.43 months (95% CI was not reached), the mPFS was 11.60 months (95% CI, 7.46-15.74).

**Figure 3:**
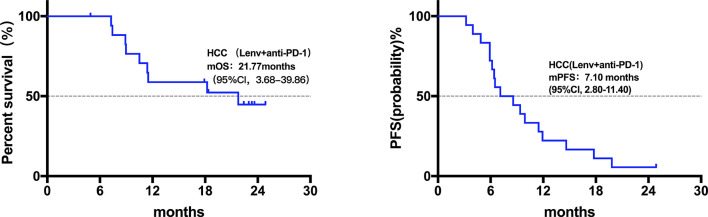
Kaplan-Meier estimates of overall and progression-free survival in HCC patients treated with anti-PD-1 combine with Lenvatinib. The mOS of HCC patients treated with anti-PD-1 combined with Lenvatinib was 21.77 months (95% CI, 3.68-39.86), mPFS was 7.10 months (95% CI, 2.8-11.4).

**Figure 4:**
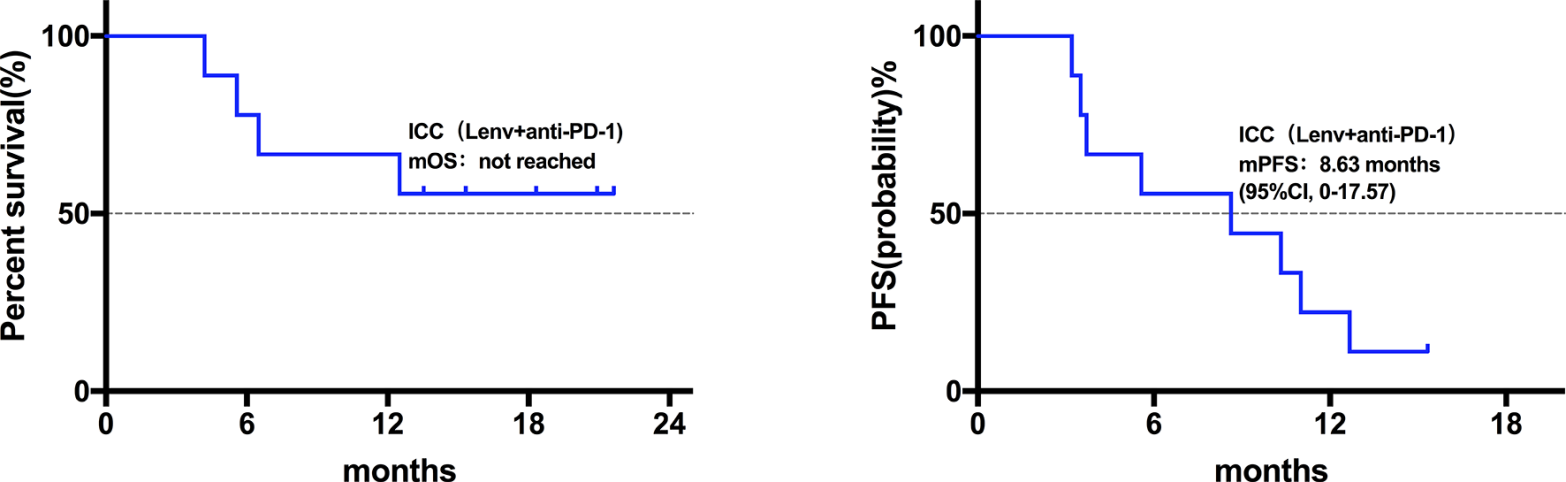
Kaplan-Meier estimates of overall and progression-free survival in ICC patients treated with anti-PD-1 combine with Lenvatinib. The ICC patients did not reach mOS; mPFS was 8.63 months (95% CI, 0-17.67).

Twenty patients died at the termination of the follow-up duration. The overall mOS was 22.43 months (95% confidence interval (CI) was not reached, range: 4.20-28.30 months, **Figure 1**). The 6-month, 1-year, and 18-month OS rates were 95.74% (95% CI, 84.01-98.92), 73.08% (95% CI, 57.45-83.74), and 63.22% (95% CI, 46.99-75.70), respectively. The overall mPFS was 8.93 months. (95% CI, 6.76-11.10, range: 3.20-28.30 months, **Figure 1**). The mOS and mPFS of HCC patients treated with monotherapy were 22.43 months (95% CI was not reached, **Figure 2**) and 11.60 months (95% CI, 7.46-15.74, **Figure 2**), respectively. In the HCC combination treatment group, the corresponding were 21.77 months (95% CI, 3.68-39.86, **Figure 3**) and 7.10 months (95% CI, 2.8-11.4, **Figure 3**), respectively. However, patients with ICC in the combination treatment group did not reach mOS (**Figure 4**); meanwhile, the mPFS was 8.63 months (95% CI, 0-17.67, **Figure 4**).

### Safety

Overall, Lenvatinib was well-tolerated (**Supplementary Table 2**). Twenty-one (43.8%) and 12 (25%) patients had elevated transaminase levels (grades 1-2 and 3-4, respectively). The administration of liver protection treatments returned transaminase levels to the normal range; other patients presented no abnormalities. Ten (20.8%) and 8 (16.7%) patients had bilirubin elevation of grades 1-2 and 3-4, respectively; 30 (62.5%) patients had decreased albumin levels of grades 1-2. Twenty-one (43.8%) and 6 (12.5%) patients had platelet decrease of grades 1-2 and 3-4, respectively. Nineteen (39.6%) and 3 (6.3%) patients had grades 1-2 and 3-4 of alkaline phosphatase increase, respectively. In addition, 16 (33.3%) patients developed rash grades 1-2; no other serious adverse events were observed. At the end of the study, 13 (27.1%) and 29 (60.4%) patients had a performance status score of 1 and 2 points, respectively; the remaining patients had a score of 0 points.

### Efficacy

**Figure 5:**
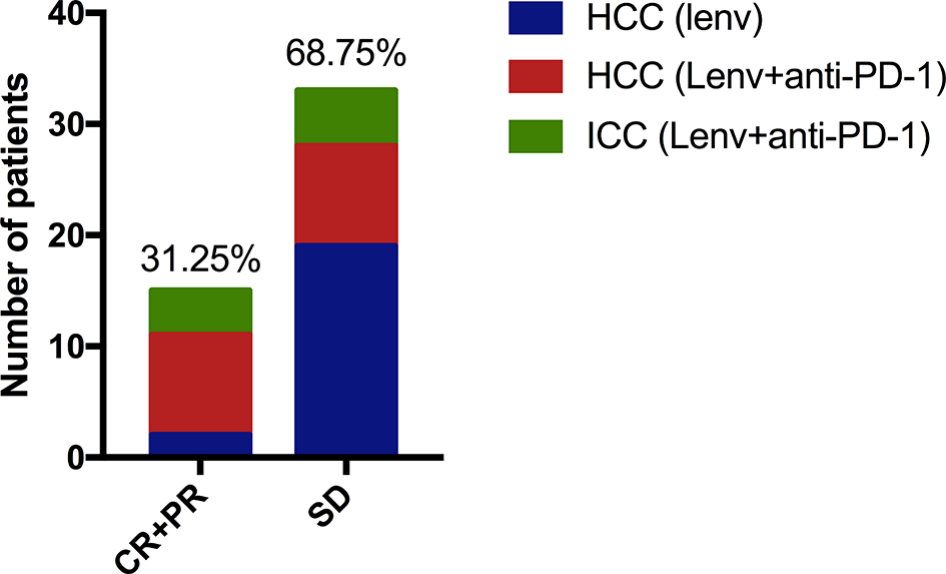
One case of HCC treated with anti-PD-1 combined with Lenvatinib achieved complete remission, and 14 patients achieved partial remission (including two HCC patients treated with Lenvatinib monotherapy. Another 4 ICC cases and 8 HCC cases were treated with anti-PD-1 combined with Lenvatinib. Thirty-three patients had SD (19 cases of HCC were treat with monotherapy, 5 cases of ICC and 9 cases of HCC received combined treatment).

One patient with HCC treated with combination therapy achieved complete response at the first review. Further, 14 patients achieved partial response (including two HCC patients treated with Lenvatinib monotherapy, and 4 and 8 ICC and HCC patients treated with combination therapy, respectively). In addition, 33 patients had stable disease (including 19 HCC patients treated with monotherapy, and 5 and 9 ICC and HCC patients, respectively, treated with combination therapy). Imaging studies revealed the overall ORR of 31.25%; the corresponding rate among the patients treated with monotherapy was 9.52%. The corresponding rates for ICC and HCC patients treated with combination therapy were 44.44% and 50.00%, respectively (**Figure 5**). The disease control rate in this study was 100%; there was no case of progressive disease at first review.

### Impact of α-Fetoprotein (AFP) and Carbohydrate Antigen 19-9 (CA19-9) Levels on Liver Cancer Prognosis

**Figure 6:**
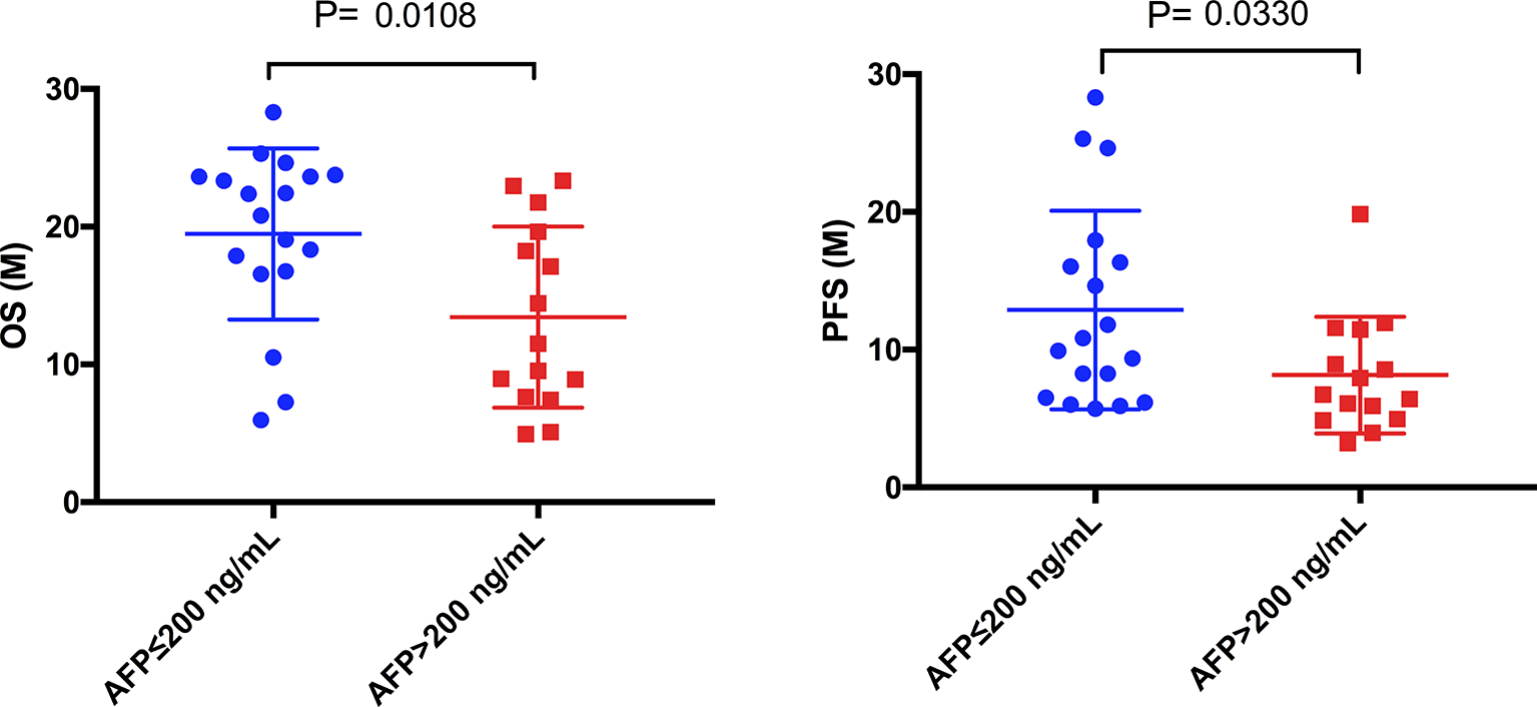
Comparison of mOS and mPFS between the low AFP and high AFP groups in HCC patients. The mOS of the low AFP group was significantly longer than that of the high AFP group (19.48 months vs. 13.43 months, P = 0.0108), as was the mPFS (12.88 months vs. 8.16 months, P = 0.0330).

Among 39 patients with HCC, 6 patients had negative baseline AFP data. We divided the remaining 33 patients into low (≤ 200 ng/mL) (n=18) and high (> 200 ng/mL) (n=15) AFP level groups. In the low AFP group, 3 and 15 patients had partial remission and stable disease, respectively; the corresponding counts for the high AFP group were 5 and 10 patients. There was no association between baseline AFP levels and treatment efficacy assessed with imaging. However, OS (**Figure 6**, P = 0.0108) and PFS (**Figure 6**, P = 0.0330) in the low AFP group were greater than those in the high AFP group. There was no association between baseline CA19-9 level of HCC patients and clinical efficacy or prognosis in the present study.

**Figure 7:**
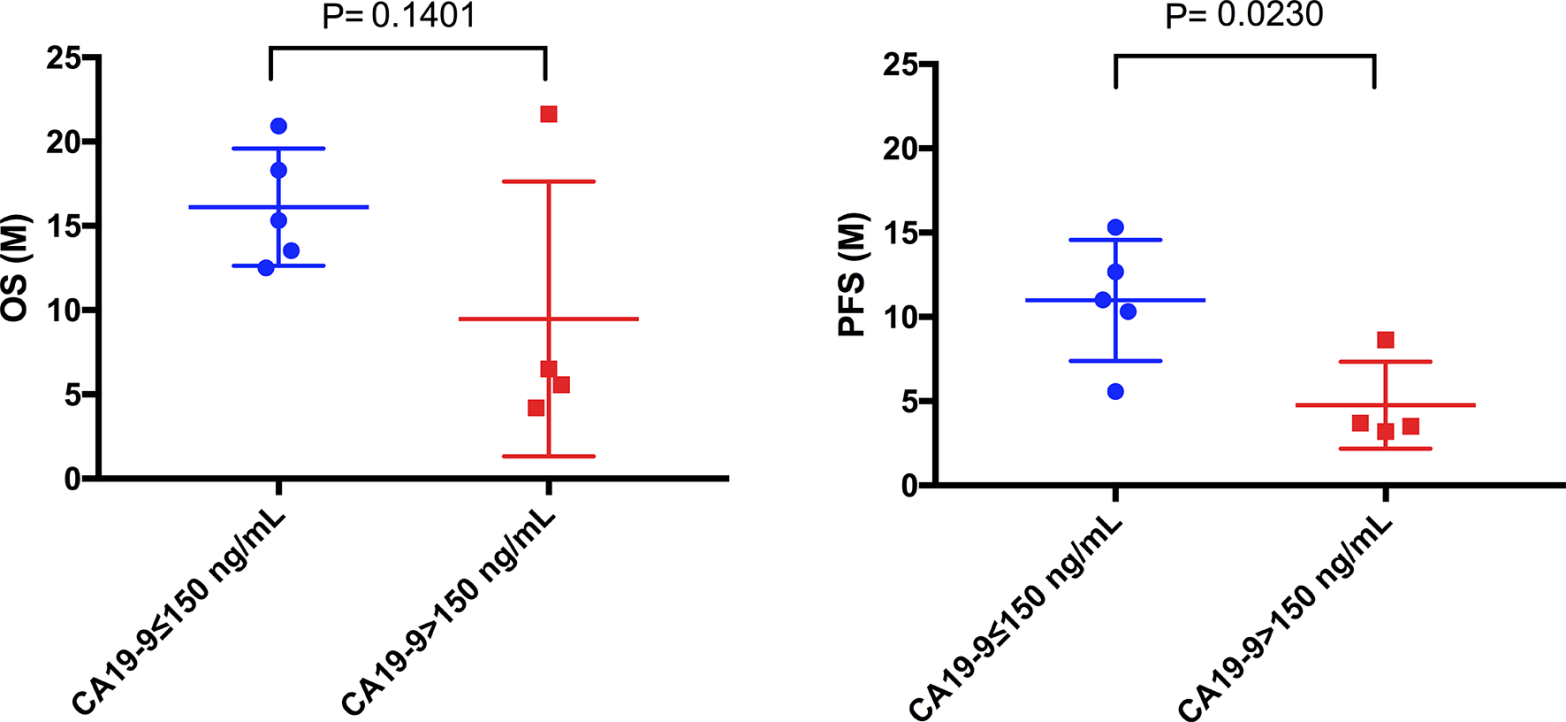
Comparison of mOS and mPFS between the low CA19-9 and high CA19-9 groups in ICC patients. The mPFS of the low CA19-9 group was significantly longer than that of the high CA19-9 group (10.78 months vs. 4.76months, P = 0.0230), but there was no significant difference in mOS (16.12months vs. 9.48 months, P = 0.1401).

In addition, we found that in 9 ICC patients, the OS between patients with high CA-199 (>150 ng/mL) (n=4) and low CA-199 (≤150 ng/mL) (n=5) has no significant difference (**Figure 7**, P=0.1401). However, PFS was significantly different (**Figure 7**, P=0.0230).

## Discussion

Lenvatinib was approved in 2018 as a first-line treatment for advanced HCC (6). This study used real-world data to assess the clinical efficacy of Lenvatinib used with or without anti-PD-1. The present ORR of patients with HCC conducted with Lenvatinib monotherapy was lower than that reported by the REFLECT trial; however, both mOS (22.43 vs. 13.6 months) and mPFS (11.60 vs. 7.3 months) in the present research were greater than those reported by the REFLECT trial (7). However, the present mOS of combined therapy were similar to those reported by the KEYNOTE-524 trial (14), which used Lenvatinib plus pembrolizumab (an anti-PD-1 antibody) in the treatment of unresectable liver cancer (21.77 vs. 22 months). The present ORR were comparable to those reported by the KEYNOTE-524 study (50.00% vs. 36%) (14). The mPFS (7.10 vs. 8.6 months) was slightly shorter than that previously reported, which may be attributed to the later disease condition of patients treated with combination therapy than that of those treated with monotherapy.

In the present study, nine patients with ICC did not reach mOS, and their mPFS was slightly greater than that previously reported by Zhao et al. (8.63 vs. 4.9 months); the present ORR was also higher than that previously reported by these authors (44.44% vs. 25%) (16). These findings suggest that PD-1 combined with Lenvatinib may benefit patients with ICC. However, the number of ICC patients included in this study was small and the follow-up time was short. In order to make our results more credible, we will enroll more patients for analysis and extend the follow-up time of patients.

The quality of life is becoming the focus of oncology research. The incidence of serious adverse events in clinical trials of sorafenib was 52% (4); the rates associated with Lenvatinib were comparable to those associated with sorafenib (7). In phase III clinical REFLECT trial, a 12-mg therapeutic dose was used for patients weighing >60 kg; in contrast, patients weighing ≤60 kg were given a dose of 8 mg (7). Reducing the dose may delay the onset of treatment-related adverse events; however, it may affect efficacy (13). In the present study, given disease stage, large tumor burden, and the overall poor liver function of the included patients, the dose of 8 mg was used. At the end of the follow-up period, patients with body weight of > 60 kg had slightly poorer mOS and mPFS than did those with body weight of ≤60 kg; however, this difference was not statistically significant. In the present study, the 8 mg dose did not affect treatment efficacy especially in the combined therapy group. However, further studies are required to validate the present findings and elucidate the relationship between drug dosage and treatment efficacy and safety.

HCC is a complex disease with multiple pathogenic mechanisms and associated with multiple risk factors; the use of a single biomarker is insufficient for prognostication (17). AFP is the most widely used and recognized serum marker in this context. Zhang found that AFP- and ultrasound-based screening every 2 years may reduce HCC mortality by 37% (18). However, this approach remains controversial. In the present study, baseline AFP levels correlated with survival, including the mOS of HCC patients; this findings is consistent with that of the REFLECT trial. However, the associated mechanism remains unclear and further research is needed. In addition, CA19-9 is a biomarker for the diagnosis of cholangiocarcinoma (19, 20). We found that CA19-9 may have a certain relationship with the prognosis of ICC. However, due to the low incidence of cholangiocarcinoma, our center currently collects very little data, and we would expand the sample size to further support our conclusion.

At present, there are many clinical studies on lenvatinib and anti-PD-1 in the treatment of advanced liver cancer malignancies, such as NCT02579616 (21), NCT03006926 (14), NCT04044313 (22), NCT03895970 (16). There are also some retrospective analysis of the efficacy of lenvatinib in the real world (23–26). In our central study, based to the limited data available so far, it was found that Lenvatinib alone or in combination with anti-PD-1 could be an effective treatment for unresectable HCC and ICC. Moreover, baseline AFP and CA19-9 levels may contribute to predict the prognostication. However, the relationships among body weight and Lenvatinib dose and toxicity require further studies. Of course, in the future work, we will expand the sample size and extend the follow-up time to collect more data to make our conclusion more credible.

## Data Availability Statement

The raw data supporting the conclusions of this article will be made available by the authors, without undue reservation.

## Ethics Statement

The studies involving human participants were reviewed and approved by Ethics committee of Comprehensive Cancer Center of Drum Tower Hospital of Nanjing University. The patients/participants provided their written informed consent to participate in this study.

## Author Contributions

SZ, JShe, and BL conceived and designed the experiments. SZ, CL, YD, JShe, and JSha performed the experiments and analyzed the samples. SZ, CL, YD, and JSha analyzed the data. SZ, JShe, and BL wrote the manuscript. All authors interpreted the data, critically revised the manuscript for important intellectual contents and approved the final version.

## Funding

This study was supported by National Natural Science Foundation of China (No. 81902914); Jiangsu Provincial Medical Youth Talent (No. QNRC2016043); and the Key Medical Science and Technology Development Project of Nanjing (No. ZKX16032).

## Conflict of Interest

The authors declare that the research was conducted in the absence of any commercial or financial relationships that could be construed as a potential conflict of interest.

The handling editor HL declared a shared parent affiliation with the authors at the time of the review.

## Publisher’s Note

All claims expressed in this article are solely those of the authors and do not necessarily represent those of their affiliated organizations, or those of the publisher, the editors and the reviewers. Any product that may be evaluated in this article, or claim that may be made by its manufacturer, is not guaranteed or endorsed by the publisher.
