# Supplementary material for: A Retrospective Study of Lenvatinib Monotherapy or Combined With Programmed Cell Death Protein 1 Antibody in the Treatment of Patients With Hepatocellular Carcinoma or Intrahepatic Cholangiocarcinoma in China
Source: Front Oncol. 2021 Dec 17;11:788635. doi: 10.3389/fonc.2021.788635 (PMC8718677; doi:10.3389/fonc.2021.788635)
Supplement: Supplementary file 1 [file Table_1.doc]

Table 1 Patient characteristics of hepatocellular carcinoma

|  | Lenvatinib (n=21) | Lenvatinib+anti-PD-1 (n=18) | Total (n=39) |
| --- | --- | --- | --- |
| Age, years; |  |  |  |
| average (range) | 63.71 (40-84) | 57.39 (32-79) | 60.70 (32-84) |
| Age group, years |  |  |  |
| <65 | 11 (52.38%) | 13 (72.22%) | 24 (61.54%) |
| 65-74 | 6 (28.57%) | 4 (22.22%) | 10 (25.64%) |
| ≥75 | 4 (19.05%) | 1 (5.56%) | 5 (12.82%) |
| Sex |  |  |  |
| Male | 18 (85.71%) | 15 (83.33%) | 33 (84.62%) |
| Female | 3 (14.29%) | 3 (16.67%) | 6 (15.38%) |
| Weight, kg |  |  |  |
| ≤60 | 11 (52.38%) | 7 (38.89%) | 18 (46.15%) |
| >60 | 10 (47.62%) | 11 (61.11%) | 21 (53.85%) |
| Child-Pugh class |  |  |  |
| A | 20 (95.24%) | 16 (88.89%) | 36 (92.31%) |
| B | 1 (4.76%) | 2 (11.11%) | 3 (7.69%) |
| Macroscopic portal vein invasion, extrahepatic spread, or both |  |  |  |
| Yes | 17 (80.95%) | 16 (88.89%) | 33 (84.62%) |
| No | 4 (19.04%) | 2 (11.11%) | 6 (15.38%) |
| Microvascular invasion |  |  |  |
| Yes | 21 (100%) | 18 (100%) | 39 (100%) |
| No | 0 | 0 | 0 |
| Hepatitis B |  |  |  |
| Yes | 18 (85.71%) | 17 (94.44%) | 35 (89.74%) |
| No | 3 (14.29%) | 1 (5.56%) | 4 (10.26%) |
| Cirrhosis |  |  |  |
| Yes | 15 (71.43%) | 16 (88.89%) | 31 (79.49%) |
| No | 6 (28.57%) | 2 (11.11%) | 8 (20.51%) |
| Barcelona Clinic Liver Cancer stage |  |  |  |
| B (intermediate stage) | 4 (19.05%) | 2 (11.11%) | 6 (15.38%) |
| C (advanced stage) | 17 (80.95%) | 16 (88.89%) | 33 (84.62%) |
| Involved disease sites per patient |  |  |  |
| 2 | 1 (4.76%) | 2 (11.11%) | 3 (7.69%) |
| 3 | 8 (38.10%) | 1 (5.56%) | 9 (23.08%) |
| 4 | 5 (23.81%) | 10(55.56%) | 15 (38.46%) |
| ≥5 | 7 (33.33%) | 5 (27.78%) | 12 (30.77%) |
| Previous radiotherapy |  |  |  |
| Yes | 2 (9.52%) | 6 (33.33%) | 8 (20.51%) |
| No | 19 (90.48%) | 12 (66.67%) | 31 (79.49%) |
| Previous intervention |  |  |  |
| Yes | 11 (52.38%) | 11 (61.11%) | 22 (56.41%) |
| No | 10 (47.62%) | 7 (38.89%) | 17 (43.59%) |
