# Supplementary material for: A Retrospective Study of Lenvatinib Monotherapy or Combined With Programmed Cell Death Protein 1 Antibody in the Treatment of Patients With Hepatocellular Carcinoma or Intrahepatic Cholangiocarcinoma in China
Source: Front Oncol. 2021 Dec 17;11:788635. doi: 10.3389/fonc.2021.788635 (PMC8718677; doi:10.3389/fonc.2021.788635)
Supplement: Supplementary file 2 [file Table_2.doc]

Table 2 Incidence of Drug-Related Adverse Events

| Toxicity parameters | Toxicity: No. of patients (%) | |
| --- | --- | --- |
| Laboratory tests | Grade 1-2 | Grade 3-4 |
| Elevated transaminases | 21 (43.8%) | 12 (25.0%) |
| Hyperbilirubinemia | 10 (20.8%) | 8 (16.7%) |
| Hypoalbuminemia | 30 (62.5%) | 0 |
| Decreased platelet counts | 21 (43.8%) | 6 (12.5%) |
| Elevated alkaline phosphatase | 19 (39.6%) | 3 (6.3%) |
| Worsening of PS by 2 points | 0 | 0 |
| Rash | 16 (33.3%) | 0 |
